# Association Between Temporal Asymmetry and Muscle Synergy During Walking With Rhythmic Auditory Cueing in Survivors of Stroke Living With Impairments

**DOI:** 10.1016/j.arrct.2022.100187

**Published:** 2022-02-24

**Authors:** Naomichi Mizuta, Naruhito Hasui, Yuki Nishi, Yasutaka Higa, Ayaka Matsunaga, Junji Deguchi, Yasutada Yamamoto, Tomoki Nakatani, Junji Taguchi, Shu Morioka

**Affiliations:** a Department of Neurorehabilitation, Graduate School of Health Sciences, Kio University, Nara, Japan; b Department of Therapy, Takarazuka Rehabilitation Hospital (SHOWAKAI Medical Corporation), Takarazuka, Japan; c Department of Rehabilitation, Nakazuyagi Hospital (HIMAWARIKAI Medical Corporation), Tokushima, Japan; d Neurorehabilitation Research Center, Kio University, Nara, Japan

**Keywords:** Cues, Central pattern generators, Gait disorders, Hemiplegia, Rehabilitation, Stroke, Walking, CWS, comfortable walking speed, EMG, electromyogram, FMA, Fugl-Meyer assessment, NNMF, nonnegative matrix factorization, RAC, rhythmic auditory cueing, VAF1, variance accounted for by 1 synergy

## Abstract

**Objective:**

To examine the relationship between temporal asymmetry and complexity of muscle synergy during walking using rhythmic auditory cueing (RAC) and the factors related to changes in muscle synergy during walking with RAC in survivors of stroke.

**Design:**

Cross-sectional study.

**Setting:**

Wards at 2 medical corporation hospitals.

**Participants:**

Forty survivors of stroke (N=40; mean age, 70.4±10.3 years; time since stroke, 72.2±32.3 days) who could walk without physical assistance.

**Interventions:**

Not applicable.

**Main Outcome Measures:**

The participants were assessed in a random block design under 2 conditions: comfortable walking speed (CWS) and walking with RAC. Single-leg support time, kinematics, and electromyograms were measured. Factors related to the complexity of muscle synergy (variance accounted for by 1 synergy [VAF1]) between the walking conditions were examined using hierarchical multiple regression analysis.

**Results:**

In the RAC condition, lower limb flexion and knee flexion angles, single-leg support time on the paretic side, and the symmetry index of single-leg support time were increased compared with those in the CWS condition. VAF1 was decreased in the RAC condition (73.9±0.15) compared with that in the CWS condition (76.9±0.13, *P*=.002). Hierarchical multiple regression analysis revealed that the change in VAF1 was explained by change in single-leg support time (*R*^2^=0.43, *P*=.002).

**Conclusions:**

The RAC condition demonstrated a more complex representation of muscle synergy than the CWS condition; the change in single-leg support time on the paretic side related to the changes in muscle synergy more than changes in lower limb angle. These findings can help in the walking-training concept to improve muscle synergy deficits in survivors of stroke.

## HIGHLIGHTS

• We examined the effect of temporal gait asymmetry on muscle synergy post stroke.
• In our design, the temporal asymmetry during gait was experimentally modulated.
• The temporal asymmetry was modulated using rhythmic auditory cueing.
• Rhythmic auditory cueing with gait immediately improved temporal asymmetry and muscle synergy deficits.
• The temporal asymmetry affected muscle synergy more than kinematics.

Temporal asymmetry is a characteristic of walking disorders caused by neurologic deficits in survivors of stroke.^1^^,^^2^ Temporal asymmetry is a state in which the temporal variables (eg, stance or stance time) during walking differ between the paretic and nonparetic lower limbs. Particularly, the asymmetry in single-leg support time is strongly related to motor paralysis, sensory deficits, and spasticity severity.^3^^,^^4^ However, the degree of temporal asymmetry deficits varied among survivors of stroke, which is considered to be affected by the voluntary and automatic control of walking.^5^^,^^6^ In particular, excessive voluntary control is related to the asymmetry of single-leg support time.^5^^,^^6^

Automatic control of walking is performed by subcortical functions, and efficient walking can be reproduced by controlling muscle synergy.^7^, ^8^, ^9^ Muscle synergy is the underlying cooperative pattern structure in highly variable muscle activation patterns inherent in the central nervous system.^8^^,^^9^ In a healthy person without stroke, 4 independent muscle synergies control the movement of the lower limbs during relaxed walking.^7^^,^^8^ In contrast, the independent activation of muscle synergy is impaired in many survivors of stroke, and consequently, the muscle synergies are merged (monotonic activation of muscle synergy).^8^^,^^10^, ^11^, ^12^ This monotonous muscle synergy is associated with asymmetry in the kinematics and spatial parameters of walking in survivors of stroke in some previous studies^8^^,^^11^, ^12^, ^13^; however, the relationship between temporal asymmetry of walking and complexity of muscle synergy has not been demonstrated. Because temporal asymmetry while walking in survivors of stroke is associated with impaired timing of lower limb muscle activity,^14^ it may be associated with muscle synergy, which controls the lower limb muscle activity during walking.^15^ Furthermore, because motor commands from the central nervous system control the weighting and timing for individual muscles as muscle synergy patterns,^7^^,^^8^ it is possible that temporal asymmetry relates to muscle synergy patterns. Specifically, extensor synergy modulates flexor synergy based on sensory feedback^16^; therefore, the alteration of stance duration on the paretic side is considered to significantly relate to muscle synergy. Previous studies examining the relationship between walking asymmetry and muscle synergy had a cross-sectional design and did not experimentally modulate the factors^8^; therefore, the relationship is unclear. Hence, temporal asymmetry during walking should be experimentally modulated to clarify its relationship with muscle synergy. Clarifying the mechanism of muscle synergy impairment during walking in survivors of stroke provides important insights for walking rehabilitation for them.

A simple method to improve walking asymmetry in survivors of stroke is rhythmic auditory cueing (RAC).^17^ RAC postulates that temporal symmetry could be manipulated as a conditional setting because synchronizing the timing of foot contact with a metronome tempo during walking immediately improves asymmetry.^17^^,^^18^ We hypothesized that RAC during walking, as a conditioned manipulation, would increase the single-leg support time on the paretic side and provide a complex representation of muscle synergy.

This study aimed to determine the relationship between temporal asymmetry and complexity of muscle synergy during walking with RAC in survivors of stroke. Moreover, we examined the factors that relate to the alteration of muscle synergy with RAC. Clarification of the relationship between the complexity of muscle synergy and temporal asymmetry during walking suggests that interventions for temporal asymmetry may be useful in walking rehabilitation for muscle synergy deficits in survivors of stroke; additionally, training with both walking and RAC may benefit them.

## Methods

### Participants

Forty survivors of stroke (mean age, 70.4±10.3 years; time since stroke, 72.2±32.3 days) were enrolled at our hospital in this cross-sectional study (table 1). The inclusion criteria were as follows: (1) the ability to walk independently without the assistance of physical therapists, (2) the ability to walk without using walking aids with casters, (3) absence of bilateral lesions, (4) a Mini-Mental State Examination score ≥24 points, (5) absence of a history of orthopedic disease, (6) absence of pain, (7) absence of cerebellar lesions or resting tremors, and (8) absence of unilateral spatial neglect. All participants provided written informed consent before the start of the study. All procedures were approved by the Institutional Ethics Committee of the Takarazuka Rehabilitation Hospital of Medical Corporation SHOWAKAI (ethics review no.: 2019-P-2) and were performed in accordance with the Declaration of Helsinki.

**Table 1:** Demographics of participants

Age (y), mean ± SD	70.4±10.3
Sex (n), male/female	24/16
Affected side (n), right/left	32/8
Time since stroke (d), mean ± SD	72.2±32.3
Functional Ambulation Category, mean ± SD	3.58±0.78
Using assist device (n), no use/T-cane/Q-cane	29/8/3
FMS (lower limb): max=22, mean ± SD*	20.6±2.49
FMA sensory score (lower limb): max=12, mean ± SD†	9.56±3.36
Modified Ashworth Scale: max=5, median (min-max)‡	0.00 (0-3)
Trunk Impairment Scale, mean ± SD	17.9±4.17
Short Form Berg Balance Scale, mean ± SD	22.3±4.39

Abbreviation: FMS, synergy score of the Fugl-Meyer assessment.

⁎ Synergy score of the Fugl-Meyer assessment.

† Sensory score of the Fugl-Meyer assessment.

‡ To evaluate the spasticity of the ankle plantar flexor muscle, a Modified Ashworth Scale was used and evaluated on a 0-5 scale.

### Experimental setup and procedures

Participants were instructed to walk 5 times on a 10-m walkway, with a physical therapist standing nearby to reduce the risk of falling. Because walking speed is underestimated without a supplementary walkway both before and after the walkway,^19^^,^^20^ 3 m of supplementary walkway was provided both before and after the 10-m walkway, for a total of 16 m of walkway. The participants were allowed to use a cane as needed to prevent falls during assessments; however, ankle-foot-orthosis and knee-orthosis were not allowed. They were instructed to walk in 2 walking conditions: comfortable walking speed (CWS) and walking with RAC. In the RAC condition, the tempo was determined by calculating the cadence of comfortable walking before starting the measurement. The participants listened to a smartphone auditory metronome application (MetroTimer version 3.3.2^a^) in the RAC condition and were asked to match the heel contact timing to the beat of the metronome.^21^ The measurement order for each walking condition was based on a randomized block design. All walking conditions were performed at a comfortable speed, and a 10-minute practice session was conducted before the measurements. We confirmed that the modified Borg scale score was <4 before the walking measurement to ensure that fatigue did not affect walking performance. Data from video, a wireless insole foot pressure sensor, and electromyogram (EMG) were collected while walking. A video camera (sampling rate: 60Hz) recorded from the sagittal plane, and the foot pressure sensor recorded data from both legs (physical information therapy^b^; sampling rate: 100Hz). A wireless surface EMG (Delsys Trigno^c^; sampling rate: 1926Hz) was recorded from the paretic side of the tibialis anterior and soleus, medial gastrocnemius, vastus medialis, rectus femoris, semitendinosus, biceps femoris, and gluteus medius.^22^^,^^23^ The wireless EMG had a built-in accelerometer. Each skin site was shaved and cleaned with alcohol before electrode placement. Additionally, the wireless EMG with a built-in acceleration sensor was attached to the back of the third lumbar level to record trunk acceleration data.

### Data recording and analysis

The Fugl-Meyer assessment (FMA) was used to measure the severity of motor paralysis and sensory disturbances. The FMA synergy score was used to determine the FMA motor score.^6^ To evaluate muscle spasticity of the ankle plantar flexor muscle, the modified Ashworth Scale was used and converted to a 0-to-5–point scale (0: no increase in muscle tone, 5: affected part[s] rigid in flexion or extension). The Trunk Impairment Scale was used to assess trunk function, and the Short Form Berg Balance Scale was used to assess balance ability. Walking independence was assessed using the Functional Ambulation Category test.

Walking speed and cadence were measured using a stopwatch when participants passed the start and end lines of the 10-m walkway using the recorded video data. After removing the first and last 3 gait cycles from the data set, 20 strides for each participant and condition were extracted.^24^ The mean absolute percentage error was calculated to determine accuracy with respect to the metronome while walking.^21^

(1) $$Meanabsolutepercenterror=\frac{\left|Measuredcadence-RAC\right|}{RAC}*100$$

The symmetry of the single-leg support time was evaluated using the symmetry index.^25^ To calculate the joint angle, a recorded walking video was obtained using OpenPose version 1.4.0.^26^^,^^27^ The joint movement parameters were calculated as the lower limb and knee joint angles on the paretic side. The lower limb angle on the paretic side was defined as the angle between the vertical axis and the vector joining the hip joint with the ankle joint; a high value was set as the direction of flexion.^28^ The lower limb angle was analyzed based on the fact that the nervous system controls the angle of limb kinematics more than that of joint kinematics.^29^ Lower limb angle and knee joint angle data were low-pass filtered using a 0-lag fourth-order Butterworth filter with a cutoff frequency of 6 Hz.^30^ Lower limb flexion and extension angles were defined as peak angles during walking. The knee joint angle was calculated as the peak flexion angle during the swing phase. The acceleration signal was decomposed into static gravity and dynamic acceleration components to remove the confounding effects of both the gravity measurement and inevitable accelerometer misalignment.^31^^,^^32^ The signals obtained from the accelerometer were demeaned and low-pass filtered (fourth-order 0-lag Butterworth filter at 10Hz).^33^ The raw EMG signals were band-pass filtered using a 0-lag fourth-order Butterworth filter with cutoff frequencies of 20-500 Hz, demeaned, rectified, and low-pass filtered using a 0-lag fourth-order Butterworth filter with a cutoff frequency of 10 Hz. The EMG signals were normalized by dividing them by the maximal amplitude obtained during walking. All preprocessing EMG procedures were performed following the Surface Electromyography for the Non-Invasive Assessment of Muscles guidelines (http://www.seniam.org). Identification of walking events was performed based on the vertical component of the triaxial accelerometer attached to the third lumbar level and a wireless insole foot pressure sensor on both sides.^34^ Additionally, to identify joint angle walking events, the ankle joint coordinate data were differentiated twice and converted to acceleration data, and then the heel contact of both legs was determined. We adapted linear interpolation over individual gait cycles based on the timing of initial contact to fit acceleration, EMG, and joint angle data to a normalized 100-point time base.

Nonnegative matrix factorization (NNMF) was then performed to extract muscle synergies from the concatenated EMG data from each walking condition.^8^ EMG data time normalized to 100% of the gait cycle were used to calculate muscle synergy. NNMF was performed using a multiplicative update algorithm. Parameters for the tolerance for the residual were given as 1e−6 and the tolerance for the relative change in elements was given as 1e−4. The algorithm was repeated 1000 times and the results with the lowest residuals of root mean square were used.^35^ NNMF decomposes the EMG signals into 2 matrices: *W* containing the synergy weights, which are the weighted contributions of each included muscle to each synergy, and *C,* the synergy activations:

(2) $$EMG=\left(W_{m*n}*C_{n*t}\right)+error$$

In equation 2, *n* is the number of synergies, *m* is the number of muscles, and *t* is the number of data points. The *error* value between the measured EMG data and the reconstructed EMG signals from the calculated synergy was used to calculate the variance accounted for using the formula

(3) $$VAF_{n}=\left(1-\frac{\left[\sum_{j}^{t}\sum_{j}^{m}{\left(error\right)}^{2}\right]}{\left[\sum_{j}^{t}\sum_{j}^{m}{\left(EMG\right)}^{2}\right]}\right)$$

NNMF analyses were performed with the output constrained to 1 module. The total variance accounted for by 1 synergy (VAF1) was used as an estimate of the complexity of the synergy.^13^^,^^36^, ^37^, ^38^, ^39^, ^40^, ^41^ When VAF1 is high, it can explain most of the variance in muscle activation, which indicates a reduction in the complexity of motor control during the task.^36^, ^37^, ^38^^,^^40^, ^41^, ^42^ Changes in all walking parameters between walking conditions were calculated by subtracting the RAC from the parameters in the CWS condition. MATLAB R2017a was used for all data analyses.^d^

### Statistical analysis

Paired *t* tests were used to compare all the variables between the CWS and RAC conditions. The relationship between the walking parameters in the CWS condition was confirmed using Spearman rank correlation analysis. To confirm the relationship between VAF1 changes and walking parameters between conditions, Spearman rank correlation analysis was performed. Additionally, a hierarchical multiple regression analysis was conducted with the change in VAF1 as a dependent variable, and a model was created by adding the changes in lower limb flexion and extension angles and knee flexion angle in step 1 to confirm the relationship with kinematics variables. Finally, to confirm the relevance of the temporal factor, the change of single-leg support time on the paretic side was added in step 2. In these steps, variables were added manually to separate the relationships with kinematic and temporal factors of walking. All analyses were completed using R statistical software.^e^ Statistical significance was set at *P*<.05, and the values are presented as mean ± SD.

## Results

### Effects of rhythmic auditory cueing on walking parameters

**Fig 1:**
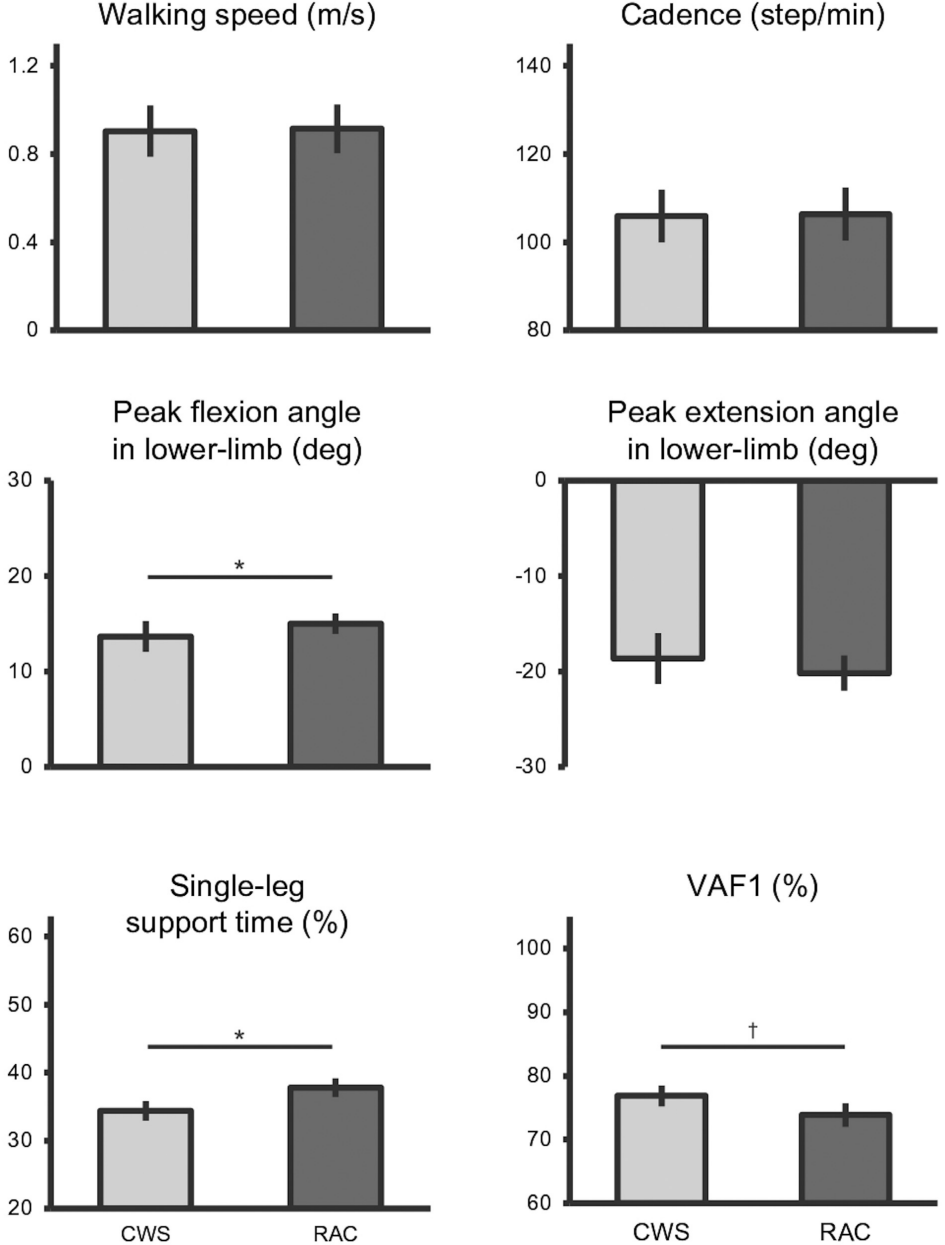
Comparison of the walking parameters between CWS and RAC conditions. The light gray bar shows the CWS, and the dark gray bar indicates the RAC condition. Data are reported as mean ± 95% CI. *P<.001. ^†^*P*<.01.

**Fig 2:**
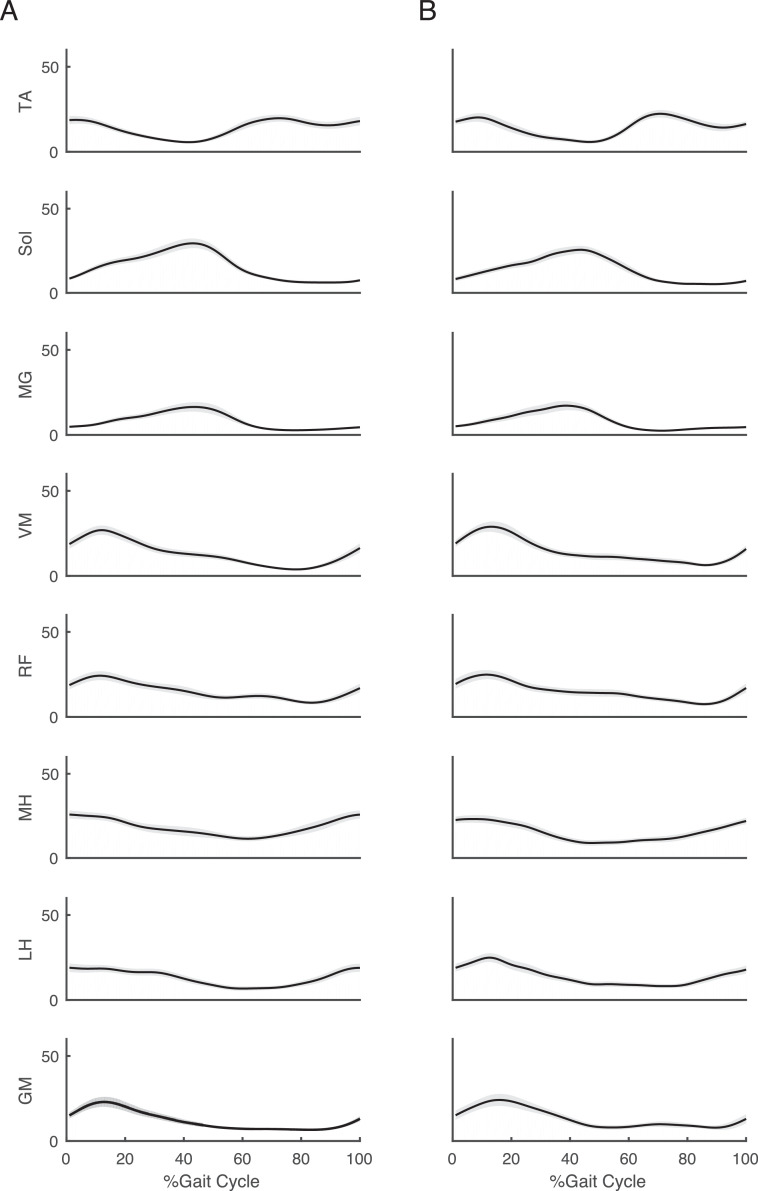
Muscle activity patterns in each walking condition. Solid black lines and gray areas represent mean ± SE. The values are given as percentages. (A) is the CWS condition, and (B) is the RAC condition. From the top to the bottom, the figure shows the TA, Sol, MG, VM, RF, MH, LH, and GM muscle activity during the 100 percent gait cycle. Abbreviations: GM, gluteus medius; LH, biceps femoris; MG, medial gastrocnemius; MH semitendinosus; RF, rectus femoris; Sol, soleus; TA, tibialis anterior; VM, vastus medialis.

A decrease in single-leg support time on the paretic side in the CWS condition was observed in many participants. All participants had low mean absolute percentage error (3.47±2.68) and walked with RAC in synchronization with the tempo setting of the metronome. The RAC condition had no effect on walking speed (*P* =.208) and cadence (*P* =.581) compared with the CWS condition. Additionally, the lower limb flexion angle (*P*<.001), knee flexion angle (*P* =.004), single-leg support time (*P*<.001), and symmetry index of single-leg support time (*P* =.006) on the paretic side were significantly increased in the RAC condition compared with the CWS condition, while the lower limb extension angle (*P* =.185) was not different (table 2). VAF1 was significantly reduced in the RAC compared with the CWS condition (*P* =.002) (fig 1). We observed slight between-condition differences in the peak timing and amplitude of muscle activation during walking (fig 2).

**Table 2:** Comparison of walking parameters between comfortable walking and rhythmic auditory cueing conditions

	Walking Conditions			95% CI			
Variable	CWS	RAC	Mean Difference	Lower	Upper	Cohen *d*	*P* Value
Walking speed	0.90±0.06	0.92±0.06	−0.011	−0.028	0.006	−0.203	.208
Cadence	105.9±2.96	106.4±2.98	−0.406	−1.88	1.069	−0.088	.581
Peak flexion angle in the lower limb	13.7±0.79	15.0±0.53	−2.106	−2.767	−1.445	−1.095	<.001*
Peak extension angle in the lower limb	−18.7±1.32	−20.2±0.90	1.399	−0.702	3.501	0.229	.185
Peak flexion angle in the knee joint	44.3±1.62	48.3±1.85	−4.124	−6.837	−1.410	−0.522	.004†
Single-leg support time	34.4±0.71	37.8±0.67	−3.422	−4.421	−2.422	−1.095	<.001*
Symmetry index in single-leg support time	−2.92±0.59	−1.78±0.46	−1.142	−1.941	−0.342	−0.456	.006†
VAF1	76.9±0.80	73.9±0.92	3.023	1.136	4.910	0.512	.002

NOTE. Data are reported as mean ± SE. The *P* value indicates a paired *t* test between the walking conditions.

⁎ *P*<.001.

† *P*<.01.

### Influencing factors of changes in VAF1 by rhythmic auditory cueing

**Fig 3:**
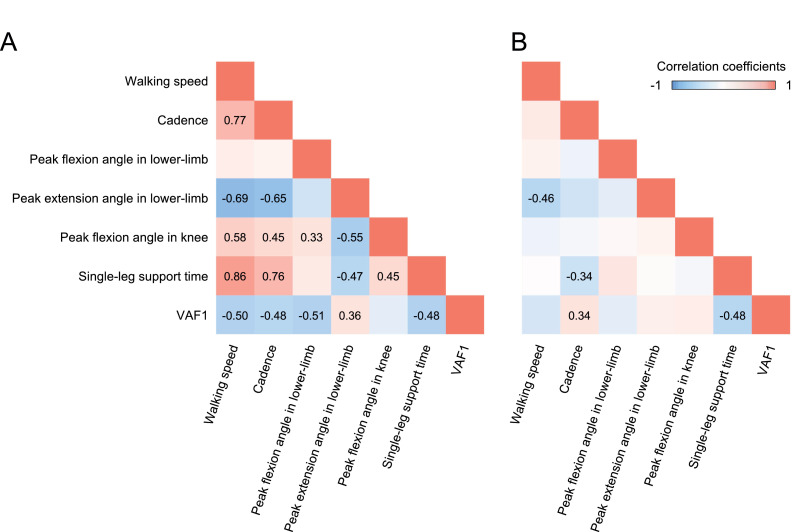
Correlation matrix of walking parameters. The matrix of Spearman rank correlation coefficients of walking parameters is shown in the color scale. The ρ value is indicated only in the pixels when the correlation was significant at *P*<.05. (A) Walking parameters in the CWS condition and VAF1 associated with walking speed, cadence, lower limb flexion, and extension angles and single-leg support time on the paretic side. (B) Changes between the CWS and RAC conditions. ΔVAF1 was associated with Δcadence and Δsingle-leg support time on the paretic side.

VAF1 in the CWS condition was related to all the walking parameters (fig 3A). Because VAF1 decreased more in the RAC condition than in the CWS condition, factors related to the change in VAF1 were confirmed; ΔVAF1 was associated with Δcadence (*r*=0.34, *P*=.031) and Δsingle-leg support time on the paretic side (*r*=−0.48, *P*=.002); however, Δwalking speed (*P*=.088), Δlower limb flexion (*P*=.317), Δextension angles (*P*=.304), and knee flexion angle on the paretic side (*P*=.247) were not associated with ΔVAF1 (fig 3B).

In step 1, to confirm the relationship between VAF1 and kinematics, Δlower limb flexion angle (*P*=.456), Δlower limb extension angle (*P*=.527), and Δknee flexion angle (*P*=.790) were entered; however, the model was not significant (*R*^2^=0.04, *P*=.738). To further examine the relationship between VAF1 and the temporal factor, when Δsingle-leg support time was included in step 2, the explanatory rate of the model increased to 38.8% from step 1 (*R*^2^=0.43, *P*=.002) (table 3).

**Table 3:** Relationship between temporal factors of walking and ΔVAF1 on hierarchical multiple regression analysis

Independent Variable	Unstandardized Coefficients	SE	Standardized Coefficients	95% CI		Collinearity Statistics		*t* Value	*P* Value	*R*^2^	BIC	*P* Value
				Lower	Upper	Tolerance	VIF					
Step 1										0.039	242.1	.738
ΔLower limb flexion angle	−0.429	0.568	−0.134	−0.495	0.227	0.990	1.010	−0.755	.456			
ΔLower limb extension angle	0.116	0.182	0.115	−0.253	0.483	0.952	1.051	0.639	.527			
ΔKnee flexion angle	0.038	0.141	0.048	−0.318	0.415	0.958	1.043	0.269	.790			
Step 2										0.427	227.6	.002*
ΔLower limb flexion angle	0.353	0.479	0.110	−0.195	0.414	0.860	1.163	0.737	.467			
ΔLower limb extension angle	0.085	0.143	0.084	−0.206	0.374	0.95	1.053	0.593	.558			
ΔKnee flexion angle	0.069	0.111	0.088	−0.201	0.377	0.955	1.047	0.623	.538			
ΔSingle-leg support time	−1.248	0.277	−0.670	−0.974	−0.366	0.862	1.160	−4.504	<.001†			

NOTE. The results of a hierarchical multiple regression analysis with ΔVAF1 as the dependent variable were reported. All independent variables were the change values between the walking conditions. Step 1 confirmed the effect of the Δkinematic variables on the paretic side; however, the model was not significant. To further examine the effect of the temporal factor, when Δsingle-leg support time on the paretic side was included in step 2, the explanatory rate of the model increased to 38.8% from step 1.

Abbreviations: BIC, bayesian information criterion; VIF, variance accounted for.

⁎ *P*<.001.

† *P*<.01.

## Discussion

This study aimed to determine the association between temporal asymmetry and muscle synergy during walking with RAC in survivors of stroke and examine the factors influencing changes in muscle synergy with RAC. RAC combined with walking immediately prolonged single-leg support time on the paretic side and improved temporal asymmetry. Additionally, muscle synergy was more complex in the RAC than in the CWS condition. Moreover, the change in single-leg support time on the paretic side was a more significant factor than the changes in kinematics during walking on hierarchical multiple regression analysis.

Temporal asymmetry is a major walking deficit that reflects neurologic phenomena in survivors of stroke.^1^^,^^2^ These persons show a decrease in the number of muscle synergies.^15^ In this study, VAF1 was reduced in the RAC condition compared with the CWS condition, indicating that RAC can provide a complex representation of muscle synergy during walking. The VAF1 values in the CWS condition were similar to those reported in a previous studies.^42^ VAF1 in RAC was approximately 3% lower than in the CWS condition; however, the relationship between temporal asymmetry and VAF1 is considered weakly relevant given the variance in a previous study.^42^ Gait asymmetry reflects the level of compensation for lower limb motor paralysis, sensory deficits, and spasticity,^3^ and the complex representation of immediate muscle synergy by walking with RAC suggests overcompensation strategies in participants who have a shorter single-leg support time.^6^ The central pattern generator in human walking is hardwired to adjust the duration of the stance phase mainly by adjusting the duration of the extensor burst rather than the flexor burst.^16^ In our results, we considered that the extended single-leg support time on the paretic side and the increased symmetry index in the RAC condition compared with that in the CWS provided a possible explanation for the increased activation of the rhythm generator. Contrarily, although kinematics affect the involvement of central pattern generator,^43^ the difference in the walking speed between the CWS and RAC conditions was set to be as small as possible, and the mean difference in the peak angles of the lower limb was <2° between the conditions in our experiment (see fig 1). Additionally, in step 1 of the hierarchical multiple regression analysis, the relationship with kinematic parameters was small because the model was not significant when including peak angle of lower limb flexion and extension and knee flexion angle.

### Study limitations

The present study had some limitations. Some participants used a cane, which partially affects the walking parameters and may have influenced our results. In addition, because this study involved measurements at only 1 time point, a prospective study design is needed to clarify our findings. Furthermore, the amplitude of muscle synergy was not normalized to the participant's maximum voluntary contraction. Nevertheless, we consider that the experimental manipulation was successful because the single-leg support time was prolonged in the RAC condition. In addition, we demonstrated that RAC immediately improved single-leg support time and temporal asymmetry on the paretic side and that these were complex expressions of muscle synergy during walking. Our findings affirm the hypothesis that RAC combined with walking increases the single-leg support time on the paretic side and a complexity representation of muscle synergy. Our findings also contribute to a walking training concept to improve muscle synergy deficits in survivors of stroke.

## Conclusions

RAC combined with walking immediately changed single-leg support time on the paretic side and improved temporal asymmetry. Moreover, the RAC condition demonstrated a more complex representation of muscle synergy than the CWS condition, and the change in single-leg support time on the paretic side was related to the changes in muscle synergy more than the changes in kinematic variables on the paretic side. Our findings suggest that temporal asymmetry intervention is useful in walking rehabilitation for muscle synergy deficits in survivors of stroke and that training with RAC is beneficial.

## Suppliers

a. MetroTimer version 3.3.2; ONYX Apps, Apple Inc. b. Physical information therapy; Reif Co, Ltd. c. Delsys Trigno; Delsys Inc.d. MATLAB R2017a; MathWorks Inc. e. R statistical software Ver.4.0.5; R Core Team.
